# Mechanical characteristics of beta sheet-forming peptide hydrogels are dependent on peptide sequence, concentration and buffer composition

**DOI:** 10.1098/rsos.171562

**Published:** 2018-03-14

**Authors:** Franziska Koch, Michael Müller, Finja König, Nina Meyer, Jasmin Gattlen, Uwe Pieles, Kirsten Peters, Bernd Kreikemeyer, Stephanie Mathes, Sina Saxer

**Affiliations:** 1 School of Life Sciences, Institute for Chemistry and Bioanalytics, University of Applied Sciences and Arts Northwestern Switzerland, Muttenz, Switzerland; 2 Department for Health Science and Technology, Cartilage Engineering and Regeneration Laboratory, ETH Zurich, Zurich, Switzerland; 3 Master Program of Protein Science and Technology, Linköping University, Linköping, Sweden; 4 Department for Chemistry and Biotechnology, Tissue Engineering, Zurich University of Applied Sciences, Wädenswil, Switzerland; 5 Department of Cell Biology, University Medicine Rostock, Rostock, Germany; 6 Institute of Medical Microbiology, Virology and Hygiene, University Medicine Rostock, Rostock, Germany

**Keywords:** self-assembling peptides (SAP), SAP hydrogel stiffness, nanofibrillar architecture, SAP hydrogel degradability

## Abstract

Self-assembling peptide hydrogels can be modified regarding their biodegradability, their chemical and mechanical properties and their nanofibrillar structure. Thus, self-assembling peptide hydrogels might be suitable scaffolds for regenerative therapies and tissue engineering. Owing to the use of various peptide concentrations and buffer compositions, the self-assembling peptide hydrogels might be influenced regarding their mechanical characteristics. Therefore, the mechanical properties and stability of a set of self-assembling peptide hydrogels, consisting of 11 amino acids, made from four beta sheet self-assembling peptides in various peptide concentrations and buffer compositions were studied. The formed self-assembling peptide hydrogels exhibited stiffnesses ranging from 0.6 to 205 kPa. The hydrogel stiffness was mostly affected by peptide sequence followed by peptide concentration and buffer composition. All self-assembling peptide hydrogels examined provided a nanofibrillar network formation. A maximum self-assembling peptide hydrogel dissolution of 20% was observed for different buffer solutions after 7 days. The stability regarding enzymatic and bacterial digestion showed less degradation in comparison to the self-assembling peptide hydrogel dissolution rate in buffer. The tested set of self-assembling peptide hydrogels were able to form stable scaffolds and provided a broad spectrum of tissue-specific stiffnesses that are suitable for a regenerative therapy.

## 1. Introduction

Degradable polymeric hydrogels display several features to act as matrices for tissue engineering such as their nanofibrillar structure, high water content, elasticity and diffusion properties for small molecules [1]. Hydrogels are often polymers of natural (e.g. collagen and chitosan) or synthetic origin (e.g. poly(ethylene glycol) and poly(vinyl alcohol)) [2]. The strength and swelling properties of the three-dimensional hydrogel networks can be adjusted by the use of different covalent binding crosslinkers such as glutaraldehyde or formaldehyde or enzymatically by transglutaminase [3–6]. Alternatively, low molecular weight gelators such as peptides, saccharides or nucleotides can be used to build up the three-dimensional gel matrix based on different molecular recognition motifs [7,8]. These recognition motifs interact by hydrogen bonding, metal chelation, π–π bonding, van der Waals forces or hydrophobic bonding resulting in dynamic fibrillar hydrogels [9]. Variations in the composition of low molecular weight gelators allow us to tune the mechanical, chemical and biological properties of the resulting hydrogels and offer the advantage to generate hydrogel libraries. Furthermore, these hydrogels exhibit interesting features such as their low minimal gelation concentration and their reversible three-dimensional network formation, allowing them to sense and respond to their environment [8,9].

Among the low molecular weight gelators, self-assembling peptides (SAPs) have gained significant interest as injectable matrices [10]. To act as a potential scaffolds for tissue engineering or regenerative therapy, SAP hydrogels should closely match the nanofibrillar architecture of naturally occurring polymers (e.g. collagen) as it is known to affect cell polarity and cell migration [11–13]. Moreover, it was demonstrated that cell behaviour such as adhesion, proliferation and differentiation can be controlled by hydrogel stiffness [14–16]. Variable SAP hydrogel stiffnesses can be obtained, for example, by the increase of peptide concentration, as reported by Schneider *et al.* [17]*.* To ensure SAP hydrogel stability during tissue regeneration, the degradation rate of SAP hydrogels, which is determined by the environmental conditions, should occur in a similar time frame. For example, within the oral cavity, degradation rates of SAP hydrogels are influenced by the ionic strength (50 mM) [18] and pH (5.8–7.4) [19] or by enzymatic or bacterial digestion.

The first commercially available SAP was designed by Zhang *et al*., named RADA16, consisting of a repeated 16 amino acids long RADA motif [20]. This SAP hydrogel is able to induce chondrogenesis of bovine marrow stromal cells [21], osteoblast proliferation and differentiation *in vitro* [22]. Furthermore, RADA16 supports bone [23] and axon regeneration [24] in animal models. Besides RADA16, other SAPs such as the β-hairpin peptide MAX1, presented by Schneider and co-workers, have been shown to support the survival, adhesion and migration of fibroblasts [25–27]. Another class of SAP is called peptide amphiphile, which was designed by Stupp and co-workers and consists of an alkyl tail and a peptide head group that become increasingly hydrophilic [28]. These amphiphile gels were shown to sustain cells without the addition of cell adhesion motifs [29].

The rationally designed SAPs used in the present study were presented by Aggeli *et al*. [30–32], and consist of 11 amino acids. These SAPs assemble depending on peptide concentration, pH and ionic strength of the buffer into beta sheet and higher ordered structures such as fibrils and fibres [31]. The formed SAP hydrogels consist of fibrils and fibres with lengths in the range of several micrometres and typical fibril widths in the range of 12–19 nm [33]. The sequence of the first designed SAP called DN1 by the Aggeli group was repeatedly modified to create a SAP library with specific physico-chemical characteristics such as hierarchical self-assembly and morphology [30]. In the study of Carrick *et al*. [33], the secondary structure, made up of hierarchically stacked anti-parallel β-sheets, and fibril morphology of different Aggeli designed SAPs such as P11-4 and P11-8 were investigated at variable conditions such as pH and ionic strength.

However, a systemic evaluation of the mechanical properties (e.g. SAP hydrogel stiffness, gelation velocity or yield point) is pending. Yet, a tailored SAP hydrogel stiffness is of great interest to meet the mechanical requirements known for the different cell types and thus to induce tissue regeneration. Based on the rational design criteria defined by Bell *et al.*, Kyle *et al.* and Maude *et al.* regarding SAP net charges (+2/−2), sequences and their effect on cytotoxicity, P11-4, P11-8, P11-13/14 and P11-28/29 were selected out of the P11-library [34–36]. Therefore, the present study analyses the mechanical characteristics of four selected β-sheet SAP hydrogels using variable concentrations and buffer compositions, in order to determine their potential as three-dimensional scaffolds for cell culture and tissue engineering.

## 2. Material and methods

### 2.1. Materials

SAPs P11-4 (sequence: CH_3_CO-QQRFEWEFEQQ-NH_2_, peptide content 95%, ammonium salt), P11-8 (sequence: CH_3_CO-QQRFOWOFEQQ-NH_2_, peptide content 84.4%, TFA salt), P11-13 (sequence: CH_3_CO-EQEFEWEFEQE-NH_2_, peptide content 78.5%, ammonium salt), P11-14 (sequence: CH_3_CO-QQOFOWOFOQQ-NH_2_, peptide content 74.6%, TFA salt), P11-29 (sequence: CH_3_CO-OQOFOWOFOQO-NH_2_, peptide content 70.7%, TFA salt), and P11-28 (sequence: CH_3_CO-QQEFEWEFEQQ-NH_2_, peptide content 89.0%, ammonium salt) were purchased from CS Bio Co. and illustrated in electronic supplementary material, figure S1. Quality control was done by high-performance liquid chromatography and mass spectroscopy. Sodium chloride (NaCl), Trizma® base and magnesium sulfate (MgSO_4_, anhydrous) were purchased from Sigma–Aldrich. Dulbecco's Modified Eagle Medium (Gibco™ DMEM) 1× medium was purchased from ThermoFisher Scientific. Artificial saliva was produced as described by Strafford *et al*. protocol using Tris (120 mM), Ca(NO_3_) (4 mM), KH_2_PO_4_ (2.4 mM). Calcium nitrate tetrahydrate (Ca(NO_3_ · 4H_2_O)) and potassium dihydrogen phosphate (KH_2_PO_4_) were purchased from Sigma–Aldrich. Dulbecco's phosphate buffered saline (PBS) solution (Sigma–Aldrich) and glutaraldehyde solution (4% in borate buffer) were purchased from Sigma–Aldrich.

### 2.2. Methods

#### 2.2.1. Peptide self-assembling

For each SAP system, buffer composition was adjusted due to the specific physico-chemical properties as demonstrated in table 1. The unary SAPs (P11-4 and P11-8) were prepared, by first dissolving the lyophilized peptide powder in 100 µl of buffer A to obtain a peptide monomer solution. To induce peptide self-assembly, 100 µl of buffer B was added to the peptide monomer solution. Complementary SAPs (P11-13/14 and P11-28/29) were dissolved separately with 100 µl of their peptide specific buffers. Afterwards, peptide pairs were mixed together 1 : 1, for example 100 µl P11-13 plus 100 µl P11-14 at equimolar concentrations to get a final volume of 200 µl P11-13/14. A final ionic strength of 140 mM and a pH of 7.2–7.4 were adjusted for P11-4, P11-13/14 and P11-28/29 using 0.1 M NaOH or 0.1 M HCl. For P11-8, a pH of 7.8–8.0 was adjusted using 0.1 M NaOH.

**Table 1.** Self-assembling peptides preparation in four biological solutions.

	peptide sequence/net charge at pH 7	Tris–NaCl/MgSO_4_	DMEM	artificial saliva
P11-4	CH_3_CO-QQRFEWEFEQQ-NH_2_	A: 0.055 M Tris, pH 8	A: H_2_O	A: H_2_O
	peptide net charge : −2	B: 0.055 M Tris; 0.192 M NaCl/MgSO_4_, pH 7.0	B: DMEM 2x, pH 7	B: 2x artificial saliva pH 7
P11-8	CH_3_CO-QQRFOWOFEQQ-NH_2_	A: H_2_O, pH 6	A: H_2_O	A: H_2_O
	peptide net charge : +2	B: 0.055 M Tris; 0.236 M NaCl/MgSO_4_, pH 9	B: DMEM 2x, pH 8	B: 2x artificial saliva pH 8
P11-13	CH_3_CO-EQEFEWEFEQE-NH_2_	0.1 M Tris; 0.052 M NaCl/ MgSO_4_, pH 8	A: DMEM 1x pH 8	A: artificial saliva 1x, pH 8
	peptide net charge : −6			
P11-29	CH_3_CO-QQEFEWEFEQQ-NH_2_			
	peptide net charge : −4			
P11-14	CH_3_CO-QQOFOWOFOQQ-NH_2_	0.055 M Tris; 0.096 M NaCl/MgSO_4_, pH 7	B: DMEM 1x pH 7	B: artificial saliva 1x pH 7
	peptide net charge : +4			
P11-28	CH_3_CO-OQOFOWOFOQO-NH_2_			
	peptide net charge : +6			

#### 2.2.2. Determination of self-assembling peptide network architecture by scanning electron microscopy

Nanofibrillar structure of SAP hydrogels was formed for scanning electron microscopy (SEM) (Zeiss SUPRA® 40VP) at a concentration of 15 mg ml^−1^ in Tris–NaCl buffer to a final ionic strength of 140 mM and a final pH of 7.2–8.0, as described in table 1. SAP hydrogels were assembled overnight. Self-assembly was apparent due to the gelation and β-sheet formation of the fibrillar structure as described by Aggeli *et al.* and Carrick *et al.* (by circular dichroism, Fourier transform infrared spectroscopy and transmission electron microscopy) and was further confirmed in the present study for P11-4 and P11-8 (see electronic supplementary material, figures S4, S6 and S7) [31,33]. SAP hydrogels were fixed with a glutaraldehyde solution (4% in borate buffer, using 0.1 ml per 0.1 ml peptide hydrogel) for 24 h. Gels were then dehydrated by increasing ethanol concentration in steps (25%, 50%, 60%, 70%, 80%, 90%, 100% ethanol) at 15 min intervals. The last step (100% ethanol) was repeated three times. Afterwards, critical point drying (Balzers Union, CPD020) was applied to stabilize the natural three-dimensional network structure. Finally, the network structures were glued on an SEM stub with carbon tape and sputter coated with 2.5 nm gold–palladium (Thermo VG Scientific, Polaron, SC7620). SEM images were obtained at 10 kV with an in lens detector at a magnification of ×50 000 and a working distance of 6.0 mm. Fibre diameters were analysed with ImageJ software. Twenty-five fibre widths were measured for each picture.

#### 2.2.3. Mechanical properties of self-assembling peptide hydrogels

Dynamic oscillatory amplitude sweeps up to 150% strain were performed at 37°C using an Anton Paar MCR301 rheometer equipped with a 10 mm diameter stainless steel parallel plate geometry at a 0.9 mm measuring gap. The amplitude sweep tests were performed to measure storage moduli (*G′*) within the linear viscoelastic region. Calculations of the gel breaking points (yield points) were performed with Matlab depending on the different peptide concentrations. Therefore, the yield strain was defined as the point where *G*′ is less than 95% of its original value. Oscillatory time sweep experiments were performed to study the gelation speed and hydrogel stiffness. Time sweeps up to 150 min measuring time using a frequency of 1 rad s^−1^ and amplitude gamma of 0.05% were used. SAP hydrogel stiffness was determined after the equilibrium of the storage modulus (*G*′) was reached (*t* = 100 min). To determine the gelation speed, the slope of increasing storage modulus over time was calculated in the interval from *t* = 5 to 10 min after placing peptide solutions onto the rheometer.

##### 2.2.3.1. Effect of peptide concentration on mechanical properties of self-assembling peptide hydrogels

To study SAP hydrogel stiffness within the linear viscoelastic region and gel breaking points according to the peptide concentrations, unary SAP hydrogels (300 µl) were prepared in vials at concentrations of 15 mg ml^−1^, 20 mg ml^−1^ and 30 mg ml^−1^ by dissolving first the lyophilized peptides in 150 µl of buffer A and then adding 150 µl of peptide-specific Tris–NaCl buffer B according to table 1. Complementary SAP hydrogels (P11-13/14 and P11-28/29) were obtained by dissolving 150 µl of P11-13 in 0.1 M Tris buffer + 0.052 M NaCl pH 8 and 150 µl P11-14 in 0.055 M Tris buffer + 0.096 M NaCl pH 7.2 (table 1). The P11-13 and P11-14 solutions were then mixed (1 : 1) to obtain 300 µl of complementary SAP hydrogel with a final ionic strength of 140 mM and a pH of 7.2. All SAP hydrogels were allowed to assemble overnight.

##### 2.2.3.2. Effect of buffer composition on mechanical properties of self-assembling peptide hydrogels

To study the influence of divalent ions and different media on SAP hydrogel stiffness, gelation speed and gel breaking points (yield points), SAPs were prepared at a concentration of 15 mg ml^−1^ in the different media, Tris–NaCl, Tris–MgSO_4_, DMEM or artificial saliva, according to table 1. All experiments were done at least in duplicate. Statistical evaluation was performed with Graphpad Prism version 6.0 based on a one-way ANOVA test followed by Tukey's multiple comparison test.

#### 2.2.4. Biodegradability of self-assembling peptide hydrogels by enzymatic and bacterial exposure

SAP hydrogel dissolution and degradation properties of the peptides in the presence and absence of neutrophilic elastase and microorganisms were measured by determining the peptide content of the supernatant. Therefore, SAPs were assembled in PBS at concentrations of 20 mg ml^−1^ for P11-4 and 15 mg ml^−1^ for P11-8, P11-13/14 and P11-28/29. The assay was performed in a 96-well plate with gel volumes of 100 µl well^−1^. 86 units ml^−1^ elastase (Innovative Research, USA) (100 µg ml^−1^) were added to the assembled peptides in PBS solution. After 24 h incubation at 37°C and 5% CO_2_, 10 µl of the supernatant was sterilely removed after 24 and 168 h and measured indirectly by monomeric peptide concentration in the supernatant using the Qubit® Protein Assay (Q33211; Thermo Fisher; fluorescence at 485/590 nm) and transferred back for the remainder of the experiment allowing peptide hydrogel degradation measurement without disturbing the hydrogel to buffer fluid balance. The measurements are based on the fact that degraded peptides cause disassembly of the fibrils [37], as assembly rules are no longer given, resulting in an increase in monomeric SAP in solution. SAP concentrations were calculated based on respective peptide standard curves. For data analysis, SAP concentrations measured in the supernatant were calculated as a percentage of the specific starting concentrations of each peptide system. Statistical analysis was done using a two-way ANOVA followed by Sidak's multiple comparison test.

The stability of the hydrogels versus bacterial degradation was tested by the exposure to the bacterial strains *Streptococcus mutans* (*S. mutans*), *Pseudomonas aeruginosa* (*P. aeruginosa*) and *Staphylococcus aureus* (*S. aureus*; all from Leibniz-Institut DSMZ GmbH). Therefore, the bacterial strains were applied (10^6^ CFU ml^−1^) on the top of each gel (20 mg ml^−1^ for P11-4 and 15 mg ml^−1^ for P11-8, P11-13/14 and P11-28/29) in their respective media (Neurobasal medium for *P. aeruginosa* (Thermo Fisher), Tryptic Soy Broth (TYSB) medium for *S. mutans* and *S. aureus*). After 1 and 7 days of incubation at 37°C, 10 µl of each supernatant was analysed subsequent to centrifugation of the samples using the Qubit® Protein Assay. Calculations were performed as described previously for the elastase degradation study. Statistical analysis was performed with Graphpad Prism version 6.0 using a two-way ANOVA followed by Dunnett's multiple comparison test.

## 3. Results and discussion

### 3.1. Nanofibrillar architecture

**Figure 1.**
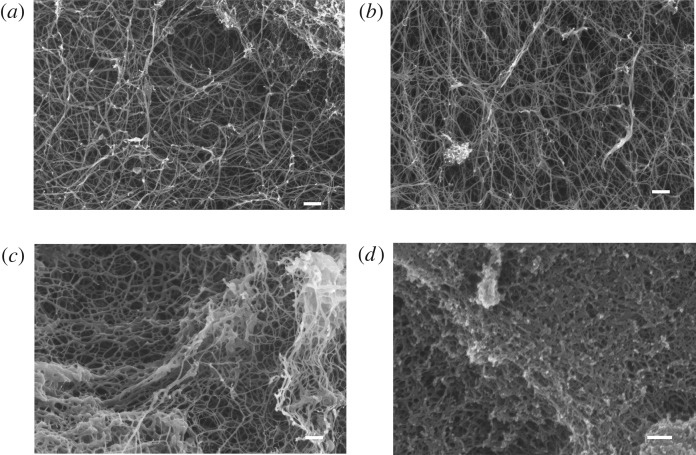
Scanning electron micrographs of self-assembling peptides in the dry state after critical point drying. (*a*) P11-4, (*b*) P11-8, (*c*) P11-13/14, (*d*) P11-28/29, magnified at ×50 000. Scale bar: 200 nm.

The topographical structure of SAP hydrogels is important for the three-dimensional arrangement of cells within scaffolds. Zhang *et al*. [38,39] state that the fibres of SAP hydrogels resemble the nanometre scale of natural polymer networks and thus allow three-dimensional cell growth. To prove the formation of nanofibrillar structure, SEM pictures were taken of dry SAP hydrogels from the four SAP sequences and compared to the literature [39] (figure 1). Quantitative analysis of fibre diameters, obtained from SEM images, revealed mean fibre widths in the following ascending order: 23 ± 4.2 nm (P11-28/29, figure 1*d*), 32 ± 6.6 nm (P11-4, figure 1*a*), 37 ± 6.6 nm (P11-8, figure 1*b*), 38 ± 10.6 nm (P11-13/14, figure 1*c*). Measured widths are at the upper level of other beta sheet peptide systems such as RADA16 with fibre diameters of approximately 10** **nm [38,40].

Carrick *et al*. [33] demonstrated that P11-4 and P11-8 prepared in NaCl formed fibril widths of 14** **nm. Moreover, they observed the formation fibre entanglements with diameters of 30** **nm. The phenomenon of fibril clustering was also reported by Zhang *et al*. [41] for the peptides KFE8 and KLD12, characterized by a typical single fibre width of 7** **nm and thicker diameters for the bundles of fibres. In alignment with the study of Leon [42] and Mishra *et al*. [43], we have also observed that the increase of SAP concentration resulted in a higher fibre density rather than in the formation of thicker fibres (see electronic supplementary material, figure S2). In addition, Branco *et al.* [44] demonstrated for MAX1 and MAX8 that the increased SAP concentration results in the formation of more fibrils that entangle and crosslink into the network. Furthermore, they draw the conclusion that higher weight per cent gels are more mechanically rigid and have smaller mesh sizes than gels of the same volume prepared with lower concentrations of peptide.

### 3.2. Mechanical properties

#### 3.2.1. Adjusting mechanical strength by self-assembling peptide concentration

**Figure 2.**
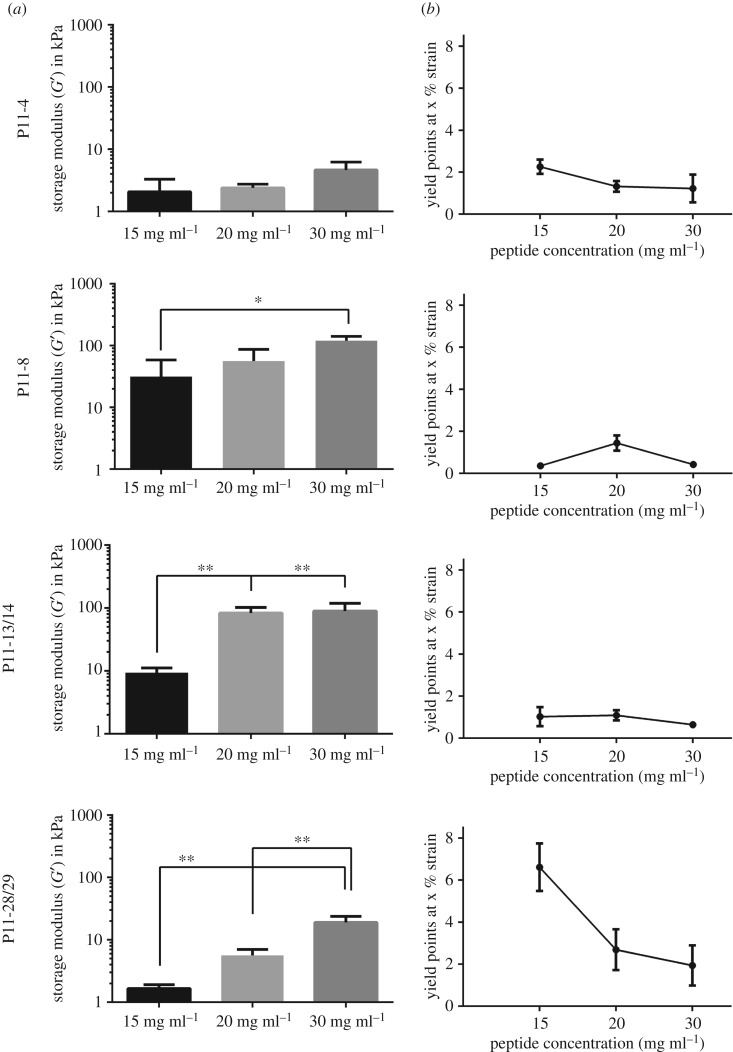
SAP hydrogel stiffnesses (*a*) in relation to the peptide concentration at 15, 20 and 30 mg ml^−1^ prepared in Tris–NaCl buffer and the influence of peptide concentration on yield points (*b*). Measurements were performed with an oscillatory amplitude sweep test on a plate to plate rheometer. *p-value ≤ 0.05, **p-value ≤ 0.01, *n* = 3.

SAP hydrogel stiffness of P11-4, P11-8, P11-13/14 and P11-28/29 prepared in Tris–NaCl (table 1) buffer at 15, 20 and 30 mg ml^−1^ was assessed by oscillatory amplitude sweep tests (figure 2*a*). For all SAPs, higher storage moduli were obtained after increasing SAP concentration from 15 mg ml^−1^ to 30 mg ml^−1^. SAP hydrogel stiffnesses ranged from low stiffness of P11-4 (2–4.6 kPa) and P11-28/29 (1.7**–**19 kPa), to high hydrogel stiffness obtained by P11-8 (31–120 kPa) and P11-13/14 (9.3–89 kPa). Furthermore, a concentration-dependent effect was found for P11-4, P11-8 and P11-28/29, whereas for P11-13/14 hydrogel stiffness did not increase further from 20 to 30 mg ml^−1^. SAP hydrogel stiffness increased by a factor of 2 (for P11-4), 4 (for P11-8) and 10 (for P11-13/14 and P11-28/29) by doubling the SAP concentration from 15 to 30** **mg ml^−1^.

The beta hairpin folding peptide MAX1 developed by Schneider *et al.* [17] resulted in storage moduli in the range of 0.04 to 2.08 kPa (factor of 52) by increasing peptide concentration from 5 to 20 mg ml^−1^ (quadrupled peptide concentration) [44]. In comparison to MAX1 at 20 mg ml^−1^, storage moduli of SAP hydrogels at 20 mg ml^−1^ used in the present study were found to be 2 to 28 times higher.

To evaluate the mechanical stability of P11-4, P11-8, P11-13/14 and P11-28/29 hydrogels, stress–strain tests were performed. Therefore, yield points, which are defined as crossover points where the hydrogel is starting to break and the material displaying fluid-like behaviour, were calculated and compared for every SAP and concentration. The earlier the yield point of a SAP hydrogel occurs, in the context of the resistance towards mechanical strain (%), the less it is tolerant towards mechanical strain. Higher hydrogel stiffness achieved by increasing SAP concentrations affects the resistance to mechanical strain as yield points decline with increasing SAP concentration (figure 2*b*). SAP concentrations of 30 mg ml^−1^ were found to have the lowest yield point, independent of the SAP sequence. The highest yield point (6.6% strain) was determined for P11-28/29 at 15 mg ml^−1^. Lowest yield points were determined for P11-8 in the range of 0.3–1.2% strain. For P11-4, yield points in the range of 1.2–2.2% strain were assessed.

As reported previously, SAP hydrogels showed low resistance to mechanical strain, approximately ≤10% [45]. For example, Goktas *et al*. [46] measured yield points below 0.5% strain for an amphiphilic peptide system and Ramachandran *et al*. [47] found that the self-assembling decapeptide system (KVW10/EVW10) did not resist strain ≥2%. Furthermore, Kirchmajer *et al.* [48] showed that the increase in genipin cross-linking of gelatin hydrogels resulted in a greater amount of elastic stiffness but reduced the extent of deformation before the hydrogel fails, which is in line with the lower yield points observed in this study.

Taken together, the increase in peptide concentration in the present study resulted in higher storage moduli (SAP hydrogel stiffness) due to the formation of more fibres at higher concentrations. The fibres exhibited additional entanglement and cross-linking into a firmer network, which led to a decrease in strain tolerance.

#### 3.2.2. Effect of ion type on mechanical properties of self-assembling peptide hydrogels

**Figure 3.**
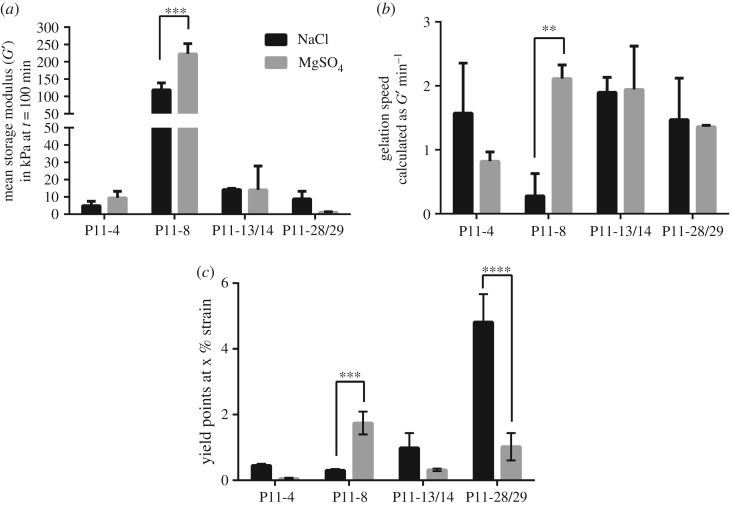
Influence of monovalent and divalent ions on SAP hydrogel stiffness (*a*), gelation speed (*b*) and yield point (*c*). SAPs were prepared either with Tris–NaCl as indicated by black bars or with Tris–MgSO_4_ indicated by grey bars. *p*-values ≤ 0.05 were defined as significant. **p-value ≤ 0.01, ***p-value ≤ 0.001, ****p-value ≤ 0.0001, *n *= 3.

Caplan *et al*. [49] demonstrated that hydrogel stiffness and the critical concentration of peptide self-assembling depend on the valence of counterions. Therefore, the influence of mono- and divalent ions on SAP hydrogel stiffness, gelation speed and yield point was analysed (figure 3). To study the effect of ion valency on the mechanical properties, SAP hydrogels were prepared with either Tris–NaCl or MgSO_4_ (table 1).

For the unary system P11-8, storage moduli increased significantly by a factor of 1.8, if SAP buffer was prepared with divalent ions such as Mg^2+^ and $\text{S}{{\text{O}}_{4}}^{2-}$ in comparison with monovalent ions such as Na^+^ and Cl^−^. Additionally, the gelation speed of, for example, P11-8 prepared with MgSO_4_ resulted in four times faster assembling time (figure 3*b*).

The increase in hydrogel stiffness and gelation speed of P11-8 prepared with MgSO_4_ was in line with an increase in resistance to mechanical strain, as the yield point increased from 0.8 to 1.6% strain (figure 3*c*). On the other hand, the preparation of P11-28/29 with MgSO_4_ resulted in a higher sensitivity to mechanical strain as the yield point declined significantly from 4.8 to 1% strain. The use of divalent ions (Mg^2+^ and $\text{S}{{\text{O}}_{4}}^{2-}$) increased storage moduli of P11-8 significantly and non-significantly for P11-4, whereas for P11-28/29 there was a decrease in hydrogel stiffness. The influence of ion valency was also observed for the SAP EAK16(II)GGH, investigated by Yang *et al*. [50], where pure beta sheet formation and extended fibre length were observed upon addition of divalent sulfate anions. These observations were explained by the salt bridge effect of $\text{S}{{\text{O}}_{4}}^{2-}$ linking two peptide molecules together.

Moreover, the type of mono- or divalent ions can further affect binding geometry and strength to the corresponding amino acid differently, as shown for glutamic acid and tryptophan by Zou *et al.* [51]. Thus for P11-15, a member of the presently investigated self-assembling peptide family, it was shown that Ca^2+^ binding site is made up of four central glutamic acid residues (two from each strand). Owing to the favourable binding energies, binding of the divalent Ca^2+^ causes a more stable fibre structure [52]. As the glutamic acid residues are also present in P11-4, and Ca^2+^ is similar to Mg^2+^, a similar behaviour can be expected for binding of Mg^2+^. The binding site is different in the complementary systems (P11-13/14 and P11-28/29) where two of the glutamates are exchanged to by ornithine leading to a less favourable binding energy and resulting in a reduced SAP hydrogel stiffness.

#### 3.2.3. Effect of ion composition on mechanical properties of self-assembling peptide hydrogels

As SAP hydrogels are dynamic systems, which respond to their environmental conditions such as ionic strength and pH by de-assembling and re-assembling, it is important to test how the mechanical properties of the four selected SAPs will be affected under biologically relevant conditions as found within the human body. Therefore, the SAP hydrogel stiffness and gelation kinetics of P11-4, P11-8, P11-13/14 and P11-28/29 were studied in serum-free DMEM and artificial saliva (table 1). Based on the observation that different ion types have an effect on hydrogel stiffness and gelation time, it was assumed that different biological media will affect also SAP hydrogel properties. Therefore, the effects of DMEM and artificial saliva, consisting of different compounds and different ion concentrations, on self-assembling kinetics, hydrogel stiffness and yield point were studied.

**Figure 4.**
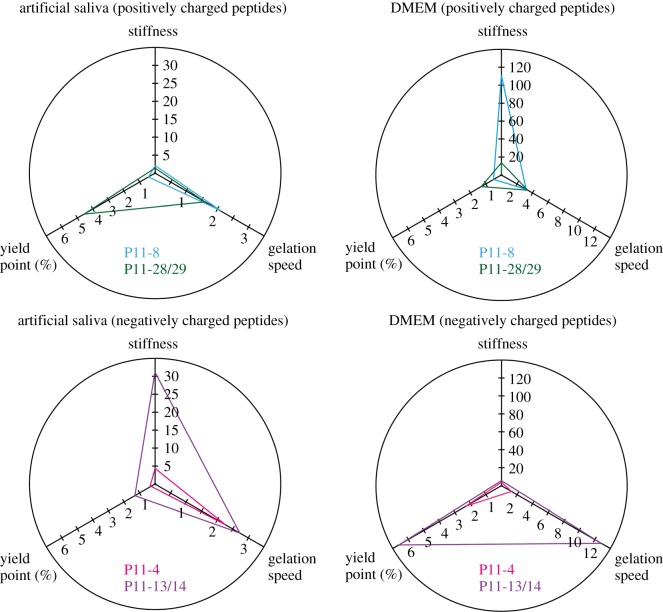
SAP hydrogel stiffness, gelation speed and yield point of P11-4, P11-8, P11-13/14, P11-28/29 as adjustable mechanical properties based on the different buffer compositions of artificial saliva and DMEM. SAP hydrogel stiffness (storage modulus *G*′ at *t* = 100 min) and gelation speed (d*G*′/d*t* 5–10 min) were determined by an oscillatory time sweep test at 0.3% strain. Oscillatory amplitude sweep experiments up to 100% strain were performed to calculate yield points, *n* = 3.

The preparation with DMEM or artificial saliva showed a significant difference in SAP hydrogel stiffness for negatively and positively charged SAP hydrogels (figure 4). In addition, there was an increase in hydrogel stiffness for SAPs with a positive overall net charge such as P11-8 (by a factor of 50) and P11-28/29 (by a factor of 9) if they were prepared with DMEM. On the other hand, hydrogel stiffness for P11-4 and P11-13/14 increased by a factor of 12 and a factor of 34, if they were prepared with artificial saliva. The gelation of both complementary SAPs P11-13/14 and P11-28/29 increased by a factor of 5 and a factor of 2 respectively, if diluted in artificial saliva.

Gelation speed increased by a factor of 1.5 for P11-8 prepared in DMEM and 1.8-fold for P11-4 in artificial saliva. For the positive complementary SAP (P11-28/29), increased gelation speed was also detected for P11-13/14 prepared in DMEM by a factor of 4.

As described in figure 3, higher storage moduli correspond to an increase in hydrogel brittleness and result in earlier yield points. This has also been observed for the different peptide preparations in biological media. For example, for P11-28/29 yield point decreases from 4.6% strain to 1.3% strain if hydrogel stiffness increased by a factor of 9, if the system was prepared with DMEM. Yield points of P11-13/14 increased from 1.3% (prepared with artificial saliva) to 6.6% (prepared with DMEM) when decreasing hydrogel stiffness by a factor of 34.

In general, both complementary SAPs (P11-13/14 and P11-28/29) were found to demonstrate a higher stability to mechanical strain (yield points in the range of 1.3–6.6%) in comparison with the unary SAPs (P11-4 and P11-8) that show yield points in the range of 0.3–2.3% strain. Moreover, an increasing hydrogel stiffness for negatively charged peptides such as P11-4 and P11-13/14 prepared with artificial saliva was observed. Ca^2+^ ions are present in artificial saliva and have a high affinity to glutamic acid [53], and we assume a similar binding site as previously described for P11-15 [52]. This is in agreement with the observations of Kirkham *et al*. [54] and Kind *et al.* [55], who demonstrated an attraction of Ca^2+^ causing crystallization of calcium phosphate from artificial saliva on P11-4 fibrillar networks.

#### 3.2.4. Testing of self-assembling peptide hydrogel stability

Stability and degradation properties of SAP hydrogels should ideally be similar to the formation rate of new tissue-specific extracellular matrix (ECM) in order to allow tissue regeneration and are thus critical parameters to be assessed [56–59]. Implanted hydrogels are exposed to inflamed tissue regions and thus to a specific mixture of cells, wound fluid, secreted enzymes and in case of an infected region also to bacteria. Thus, a preliminary test of the degradation characteristics of the four SAPs was performed after incubation with different buffers, an enzyme and bacterial strains under physiological conditions.

##### 3.2.4.1. Self-assembling peptide hydrogel dissolution in different buffer systems

**Figure 5.**
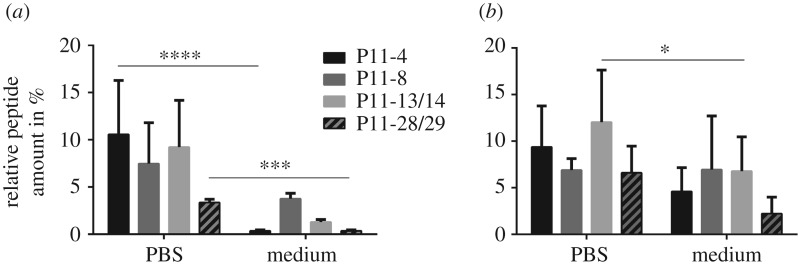
SAP hydrogel stability of P11-4, P11-8, P11-13/14 and P11-28/29 was measured by the determination of peptide concentration in the supernatant after 1 day (*a*) and 7 days (*b*) of incubation with PBS or TYSB medium. Peptide concentrations were calculated as a percentage of the peptide starting concentration, termed as % to control (*n* = 3). *p-value ≤ 0.05, ***p-value ≤ 0.001, ****p-value ≤ 0.0001.

SAP hydrogel stability was first determined by measuring SAP concentration within the supernatant after 1 and 7 days of incubation at 37°C in PBS and TYSB medium, which are characterized by different buffer capacity and buffer composition (figure 5). After 1 day incubation, SAP hydrogel dissolution of P11-4, P11-8, P11-13/14 and P11-28/29 was higher in PBS than in TYSB medium (figure 5*a*). Especially, this observation was true for P11-4 and P11-28/29 hydrogels, showing highly significant differences in PBS and TYSB SAP hydrogel stability. This effect can be explained by the different buffer composition and strength regulating the pH of the hydrogel surroundings. TYSB medium has a higher amount of K_2_HPO_4_ compared with PBS resulting in a higher buffer strength of TYSB. As the self-assembling process of the tested SAPs is known to be influenced by pH, TYSB medium keeps the pH condition stable during *in vitro* culturing when compared with PBS. Therefore, less peptide dissolution occurred in TYSB medium. Of all SAP hydrogels, P11-28/29 showed least SAP dissolution and had thus the highest SAP hydrogel stability.

##### 3.2.4.2. Self-assembling peptide hydrogel degradation by human neutrophil elastase

**Figure 6.**
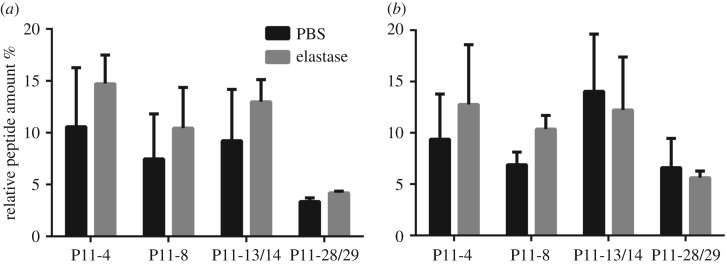
Enzymatic degradation of the unary SAP systems (P11-4, P11-8) and complementary SAP systems (P11-13/14 and P11-28/29) after 1 day (*a*) and 7 days (*b*) of incubation in PBS or in human neutrophil elastase supplemented PBS solution (100 µg ml^−1^). Peptide concentrations measured in the supernatant were calculated as a percentage of the peptide starting concentration, termed as % to control (*n* = 3).

Human neutrophil elastase represents the most abundant enzyme in inflammation-associated diseases such as diabetes [60], rheumatoid arthritis [61], cancer [62] and gingivitis [63]. It is known to cleave polyalanine (AAA) sequences [64]. SAP hydrogels were incubated with human neutrophil elastase and tested after 1 day and 7 days by measuring SAP content in the supernatant. P11-28/29 degrades two times less than the other SAP hydrogels (figure 6*a*). There was no significant difference in SAP hydrogels treated with elastase or solely PBS. The SAP hydrogels were not susceptible to elastase-mediated degradation due to the missing polyalanine sequences (AAA) of the tested SAPs. The increased standard deviation after 7 days of incubation is due to static conditions and evaporation. By comparing all four SAPs incubated solely with PBS, we conclude that the medium is the main driver which determines peptide-specific degradation rates. None of the SAP sequences have an endopeptidase cleavage site; the main degradation would, thus, be by exoproteases. Based on the insignificant SAP hydrogel degradation observed with human neutrophil elastase, it can be concluded that the gel integrity is not affected by it. In the case of a biomedical application, we assume that a specific cocktail of enzymes can digest the SAP hydrogel in short peptide fragments and single amino acids.

##### 3.2.4.3. Self-assembling peptide hydrogel degradation by bacterial strains

**Figure 7.**
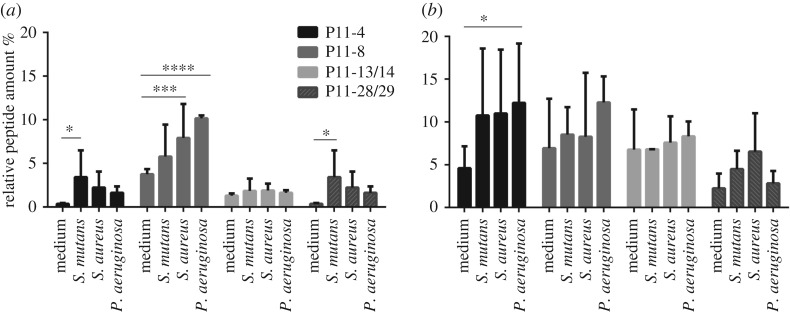
SAP hydrogel degradation after the incubation in medium and with *P. aeruginosa*, *S. aureus*, *S. mutans* was measured after 1 day (*a*) and 7 days (*b*) using 10^6^ CFU ml^−1^. The represented data were calculated in % to the peptide starting concentration (termed as % to control) of every peptide system, *n* = 3. *p*-values ≤ 0.05 were defined as significant. **p-value ≤ 0.01, ***p-value ≤ 0.001, ****p-value ≤ 0.0001.

Bacterial degradation is plausible for applications in tissue with high bacterial density such as in the oral cavity. Thus, SAP hydrogel degradation was tested after 1 day and 7 days of exposure to the three following strains: *Pseudomonas aeruginosa*, *Staphylococcus aureus* and *Streptococcus mutans*, which are common in oral cavities (figure 7). The four tested SAP hydrogels behave differently to bacterial exposure (figure 7). In the case of P11-4, P11-8 and P11-28/29, bacterial exposure affects hydrogel degradation in comparison to the medium control. Furthermore, SAP hydrogel degradation rates differ depending on the bacterial strain tested. On the other hand, no significant increase in peptide content was found for P11-13/14 after bacterial exposure. As with the enzyme degradation experiments, P11-28/29 hydrogels were degraded to a lesser extent than the other three peptide systems which were prone to be influenced by media effects. Moreover, after 7 days of exposure to bacterial strains, high standard deviations were observed due to different bacterial growth rates which led to the production of acidic metabolites and thus to a shift in pH [65]. Furthermore, the increase of data variability was caused by evaporation effects over time leading to an increase in ionic strength in the media.

In summary, the characterization of the four selected SAP hydrogels (P11-4, P11-8, P11-13/14 and P11-28/29) suggests that they are attractive candidates for a variety of tissue engineering applications due to their nanofibrillar network, adaptable stiffness by varying peptide concentration/buffer composition and degradation behaviour after enzymatic and bacterial digestion. However, it is essential, prior to clinical use, that favourable peptide candidates undergo an extensive *in vitro* evaluation with cell types specific for the selected biomedical application. Such evaluation should contain studies about cytocompatibility, inflammatory response, cell survival and adhesion as well as cell growth and differentiation capacities.

## 4. Conclusion

We have demonstrated that the SAPs (P11-4, P11-8, P11-13/14 and P11-28/29) evaluated in the present study form fibrillar networks with fibril diameters in the range of 23–38** **nm. The network architecture of the peptide hydrogels closely matched fibril diameters of the reported literature and naturally occurring ECM proteins, e.g. for collagen type І fibrils (20–200** **nm). A broad range of SAP hydrogel stiffnesses were in agreement with the stiffnesses of the different body tissue, ranging from soft (0.6 kPa) to hard (205 kPa) tissue. This stiffness variability was achieved by different assembling conditions such as peptide concentration, ionic charge and buffer composition. The self-assembling conditions were also found to affect the gelation speed and the yield point. SAP hydrogel degradation rates were mostly affected by bacterial digestion and not by enzymatic cleavage of human neutrophil elastase. Therefore, the characteristics of the SAP hydrogel environment have to be taken into account for the evaluation in future applications. Thus, we have demonstrated that the four SAPs can be modified into 48 different SAP hydrogels by varying peptide sequences, concentrations and buffer compositions. These SAP hydrogels matched the mechanical properties of soft and hard tissue stiffness and thus are potential scaffolds for regenerative therapies.

## Supplementary Material

Supplementary Information

## Data accessibility

All relevant data are within the paper and its electronic supplementary material files.
