# Supplementary material for: Mechanical characteristics of beta sheet-forming peptide hydrogels are dependent on peptide sequence, concentration and buffer composition
Source: R Soc Open Sci. 2018 Mar 14;5(3):171562. doi: 10.1098/rsos.171562 (PMC5882690; doi:10.1098/rsos.171562)
Supplement: Supplementary Information [file rsos171562supp1.docx]

**Supplementary Information**

Mechanical characteristics of beta sheet-forming peptide hydrogels are dependent on peptide sequence, concentration and buffer composition

Franziska Koch^1^, Michael Müller^2^, Finja König^3^, Nina Meyer^4^, Jasmin Gattlen^4^, Uwe Pieles^1^, Kirsten Peters^5^, Bernd Kreikemeyer^6^, Stephanie Mathes^4^ and Sina Saxer†* ^1^

1. School of Life Sciences, Institute for Chemistry and Bioanalytics, University of Applied Sciences and Arts Northwestern Switzerland, Muttenz, Switzerland.

2. Department for Health Science and Technology, Cartilage Engineering and Regeneration Laboratory, ETH Zurich, Zurich, Switzerland.

3. Master Program of Protein Science and Technology, Linköping University, Linköping, Sweden

4. Department for Chemistry and Biotechnology, Tissue Engineering, Zurich University of Applied Sciences, Wädenswil, Switzerland.

5. Department of Cell Biology, University Medicine Rostock, Rostock, Germany

6. Institute of Medical Microbiology, Virology and Hygiene, University Medicine Rostock, Rostock, Germany

†* E-mail address: sina.saxer@fhnw.ch; Phone: +41614674237.


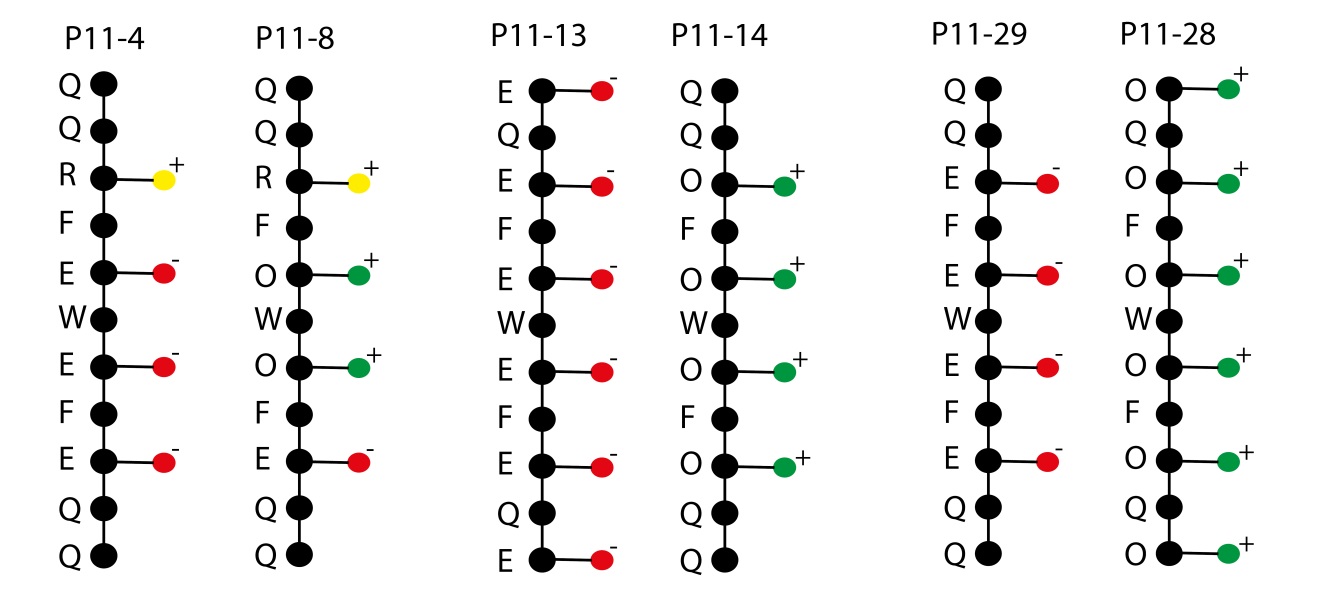


**Figure S1.** **Peptide sequences and their highlighted charged side chains**. Peptides designed by Aggeli and coworkers were renamed according to their charged side chains at physiological conditions (pH of 7.2, ionic strength of 140 mM). Black balls represent the peptide backbone, whereas red balls are used for negative charged side chains such as for carboxyl groups green and yellow balls demonstrate positive charged side chains such as amine groups.


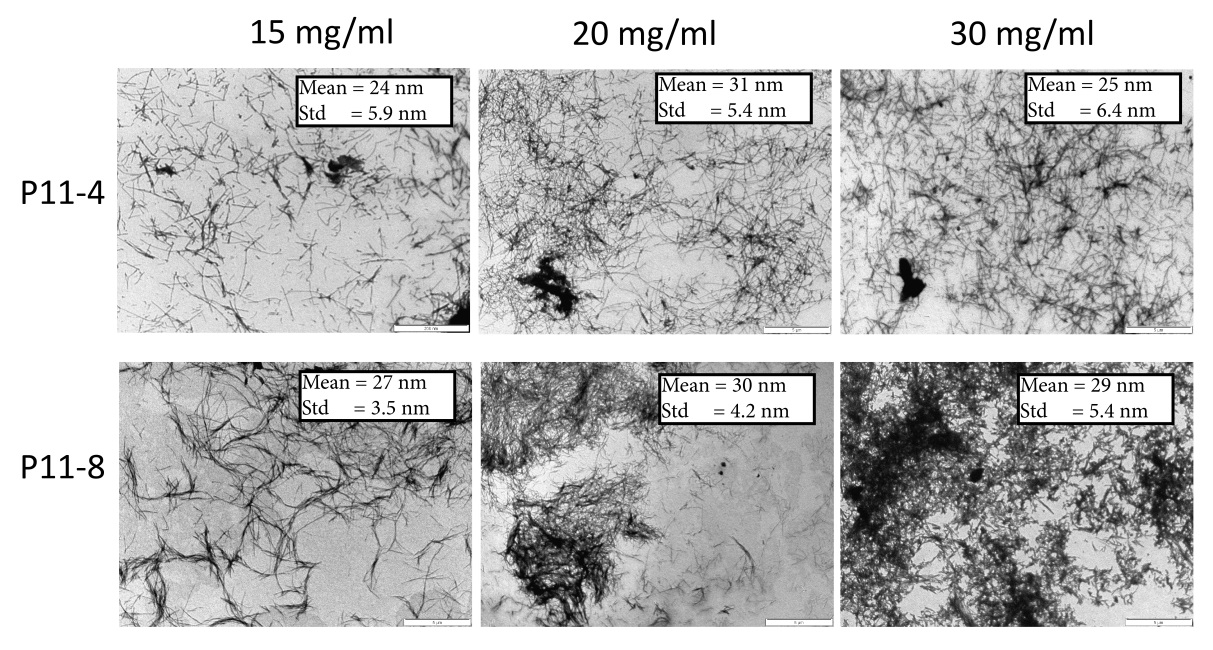


**Figure S2. Fibrillar network structures of different concentrated peptide hydrogels measured by Transmission Electron Microscopy**. The P11-4 and P11-8 peptide hydrogels were prepared at concentrations of 15 mg*ml^-1,^ 20 mg*ml^-1^ 30 mg*ml^-1^in a Tris-NaCl buffer (140mM ionic strength, pH 7.2). Samples were diluted 1:60 with water and applied to carbon-coated copper grid (200 mesh). The grids were stained with uranyl acetate solution (2% w/v in water) for 40 sec and washed twice with water and then assessed with a ZEISS EM 900 and a MegaView-III Camera (ESIS GmbH) at accelerating voltage of 80 kV. Fibre diameters were analysed with Image J software, where 25 fibre widths were measured per picture. Mean values (Mean) and standard deviations (Std) are displayed in each picture.


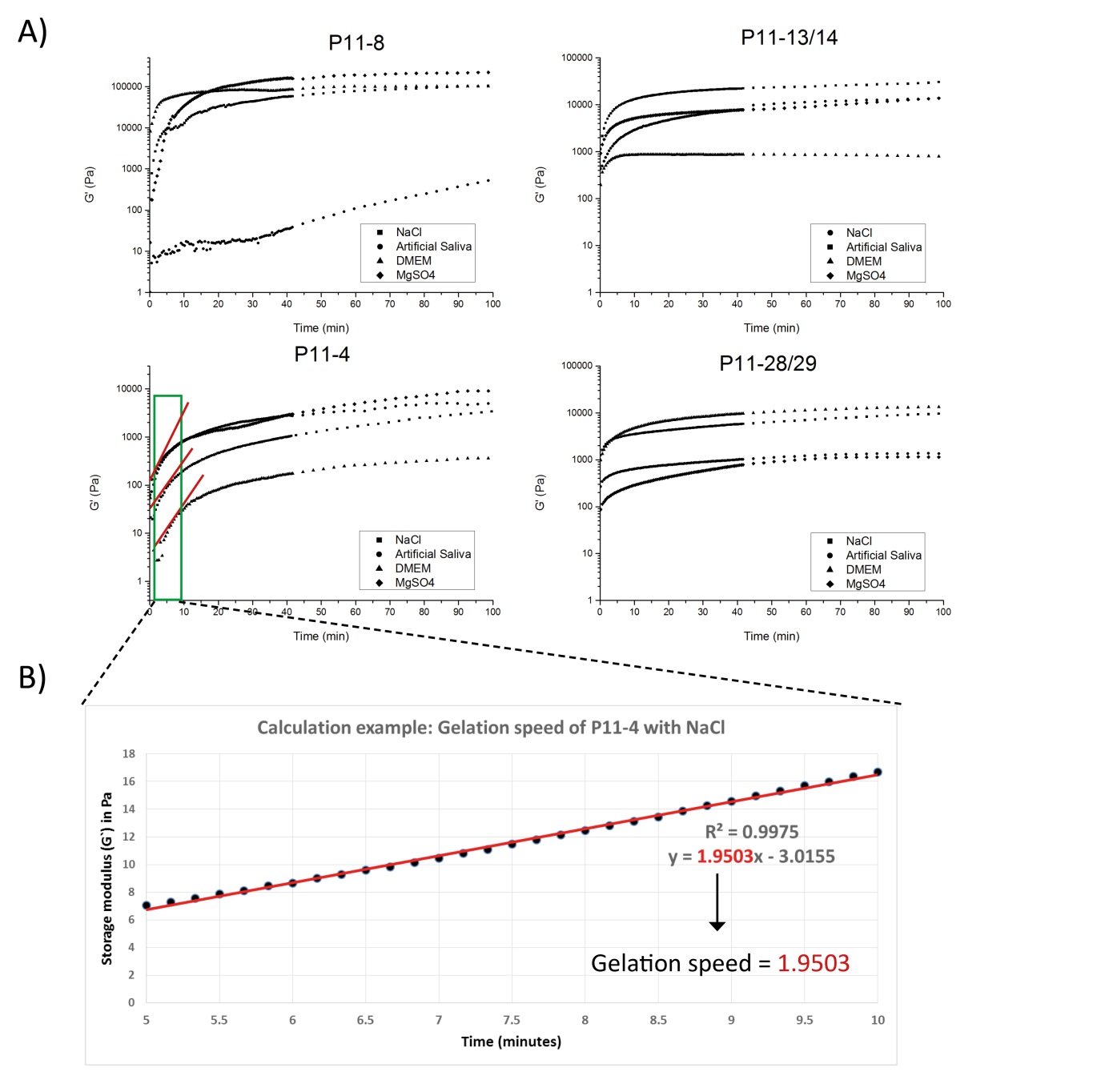


**Figure S3. Gelation speed of self-assembling peptides**. (**A**) Monitoring of storage modulus (G`) as indicator for peptide hydrogel stiffness over the time. Self-assembling peptides were prepared (15 mg*ml^-1^) with NaCl, artificial saliva, DMEM or MgSO_4_.(**B**) Example for the calculation of gelation speed within the linear area of stiffness increase (5-10 minutes). Calculations were done at least in duplicates.


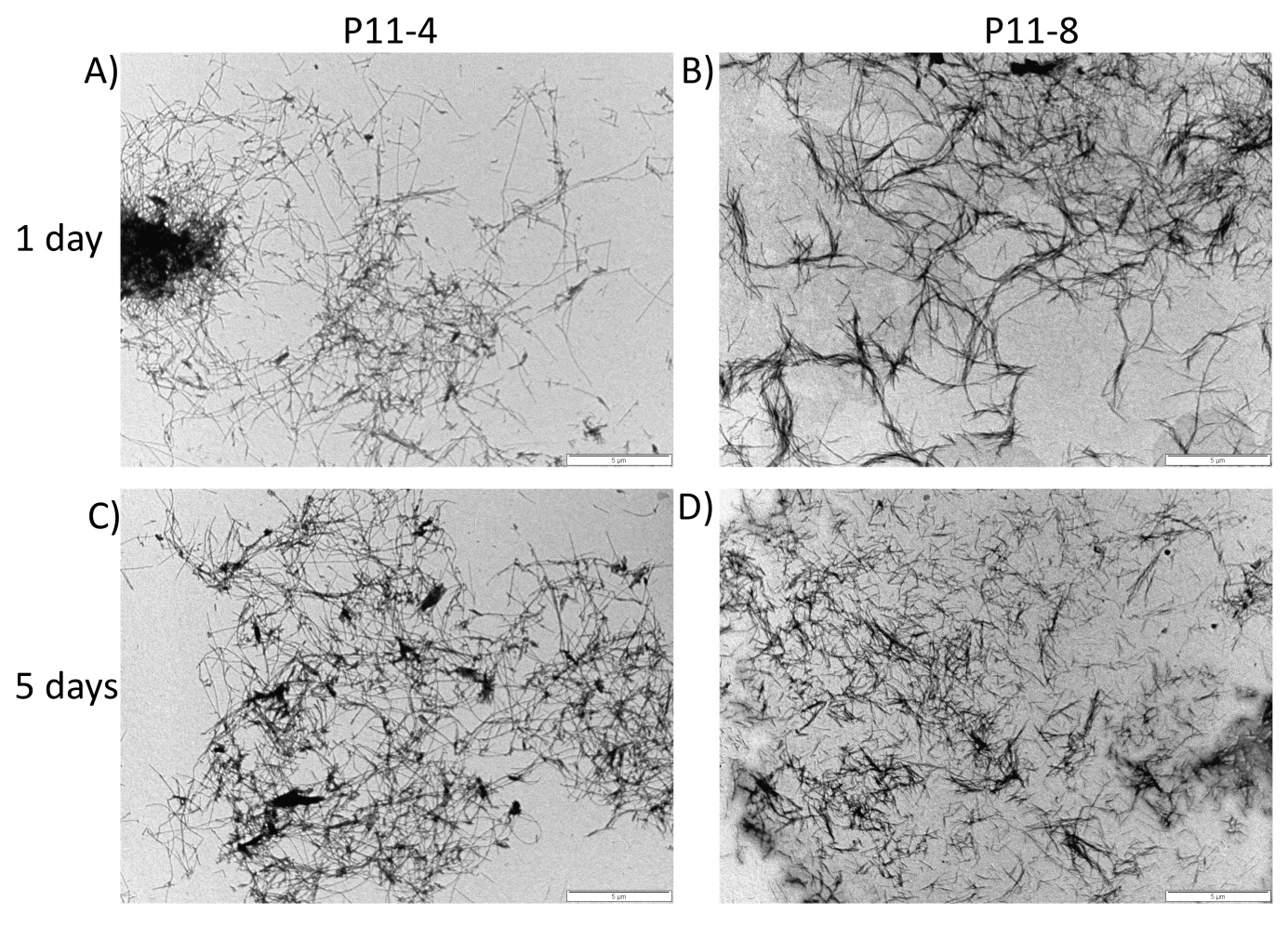


**Figure S4. Ageing of self-assembling-peptide hydrogels after 5 days measured by Transmission Electron Microscopy**. The fibrillar network structures of P11-4 and P11-8 peptide hydrogels prepared after 1 and 5 days at a concentration of 15 mg*ml^-1^_,_ in a Tris-NaCl buffer (140mM ionic strength, pH 7.2). Samples were diluted 1:60 with water and applied to carbon-coated copper grid (200 mesh). The grids were stained with uranyl acetate solution (2% w/v in water) for 40 sec and washed twice with water and were assessed with a TEM 900 (Zeiss) and a MegaView-III Camera (ESIS GmbH) at accelerating voltage of 80 kV. Scale bar is 5 µm.


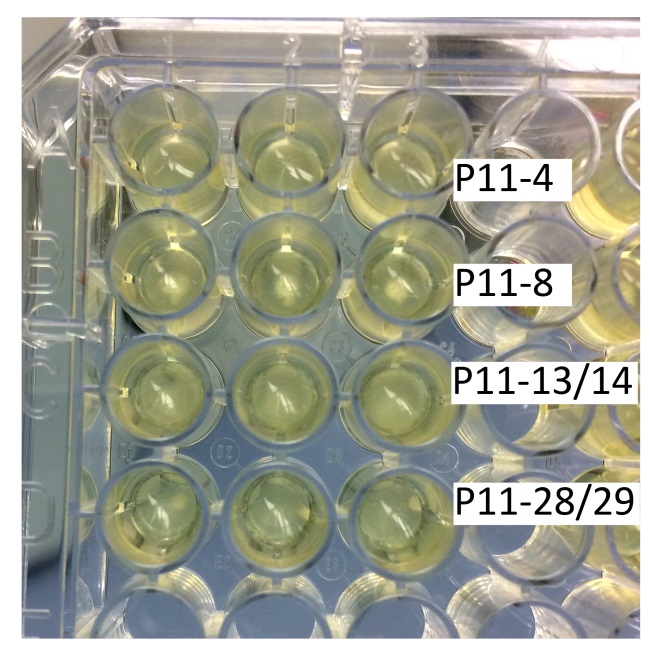


**Figure S5. SAP hydrogels after 7 days incubation with *Streptoccocus mutans*.** SAPs were assembled in PBS at concentrations of 20 mg*ml^-1^ for P11-4 and 15 mg*ml^-1^ for P11-8, P11-13/14 and P11-28/29. Bacteria were seeded on top of the gels (10^6^ CFU*ml^-1^) in Tryptic Soy Broth medium. Pictures were taken to show SAP hydrogel stability after 7 days incubation.





**Figure S6. Circular dichroism (CD) spectra of P11-4 in assembled and monomeric state**. P11-4 was prepared at 10 mg*ml^-1^ in NaCl (140 mM, pH 7.2) and assembled overnight. The red line represents antiparallel beta sheet formation of P11-4 after diluting the sample 1:63 with water. The blue line display random coil structure of P11-4 after dis-assembly of the sample which was induced by 1:63 dilution with NaF (pH 10).


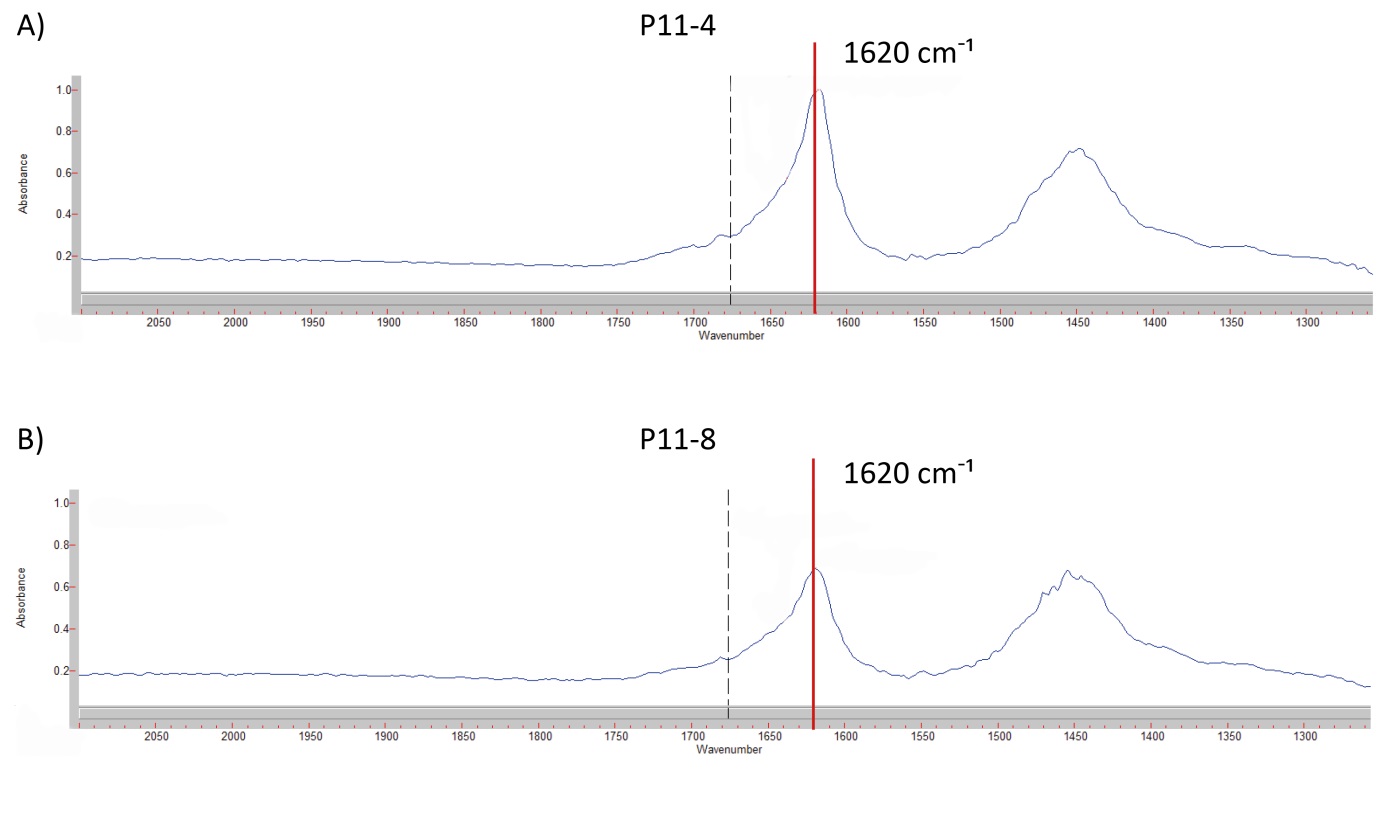


**Figure S7. FTIR spectroscopy of P11-4 and P11-8**. (A) P11-4 and (B) P11-8 hydrogels were prepared at 10 mg*ml^-1^ in NaCl in D_2_O (140 mM, pH 7.2) and analysed for beta sheet formation with FTIR (Varian 670 FTIR Spectrometer, mercurycadmium-telluride (MCT) detector,128 scans, resolution of 4 cm^–1^). FTIR absorption spectrum (peak at Amide І`1620 cm^-1^) indicates a clear beta sheet formation.

Source data:

**Table 1**: Mean peptide hydrogel stiffness (G´kPa) and yield point (%) ± standard deviation of P11-self-assembling peptides tuned by peptide concentration

|  | **P11-4** | | **P11-8** | | **P11-13/14** | | **P11-28/29** | |
| --- | --- | --- | --- | --- | --- | --- | --- | --- |
|  | Mean | YP (%) | G´(kPa) | YP (%) | G`(kPa) | YP (%) | G`(kPa) | YP (%) |
| 15 mg*ml^-1^ | 2.1± 1.0 | 2.3±0.3 | 31.6± 21.9 | 0.4±0.1 | 9.4± 1.4 | 1.0± 0.5 | 1.7± 0.2 | 6.6±1.1 |
| 20 mg*ml^-1^ | 2.4± 0.3 | 1.3±0.3 | 56.1± 25.0 | 1.4±0.6 | 83.0± 15.3 | 1.1± 0.2 | 5.6± 1.1 | 2.7±1.0 |
| 30 mg*ml^-1^ | 4.6± 1.3 | 1.2±0.7 | 120.1±16.9 | 0.4±0.1 | 89.3± 23.9 | 0.6± 0.1 | 19.2± 2.8 | 1.9±1.0 |

**Table 2:** Mean peptide hydrogel stiffness (G´ in kPa), speed (G´/min) yield point (%) ± standard deviation of P11-self-assembling peptides prepared in NaCl and MgSO_4_

|  | **P11-4** | | **P11-8** | | **P11-13/14** | | **P11-28/29** | |
| --- | --- | --- | --- | --- | --- | --- | --- | --- |
|  | NaCl | MgSO_4_ | NaCl | MgSO_4_ | NaCl | MgSO_4_ | NaCl | MgSO_4_ |
| Stiffness  (kPa) | 5.0± 2.1 | 9.5±2.7 | 119.0±14 | 222.9±21.1 | 14.3± 0.5 | 14.1± 9.7 | 8.9± 3.6 | 1.1±0.2 |
| Speed  (G`/min) | 1.6± 0.6 | 0.8±0.1 | 0.3± 0.2 | 2.1±0.1 | 1.9± 0.2 | 1.9± 0.5 | 1.5± 0.5 | 1.4±0.2 |
| YP (%) | 0.45± 0.05 | 0.05±0.03 | 0.30±0.04 | 1.74±0.35 | 0.99± 0.45 | 0.32± 0.04 | 4.82± 0.85 | 1.02±0.41 |

**Table 3:** Mean peptide hydrogel stiffness (G´ in kPa), speed (G´/min) and yield point (%) ± standard deviation of P11-self-assembling peptides prepared in artificial saliva and DMEM.

|  | **P11-4** | | **P11-8** | | **P11-13/14** | | **P11-28/29** | |
| --- | --- | --- | --- | --- | --- | --- | --- | --- |
|  | Saliva | DMEM | Saliva | DMEM | Saliva | DMEM | Saliva | DMEM |
| Stiffness  (kPa) | 4.9 ± 1.6 | 0.4± 0.01 | 2.2 ± 1.2 | 110.9 ± 9.1 | 31.2 ± 0.4 | 0.9 ± 0.1 | 1.4± 0.1 | 13.5 ± 0.3 |
| Speed  (G`/min) | 2.3 ± 0.9 | 1.3 ± 0.4 | 2.1 ± 1.0 | 3.2 ± 0.3 | 2.7± 0.2 | 12.8 ± 0.1 | 1.6 ± 0.2 | 3.3 ± 0.4 |
| YP (%) | 0.3 ± 0.02 | 2.3 ± 0.44 | 0.4 ± 0.01 | 0.5 ± 0.01 | 1.3 ± 0.43 | 6.6 ± 2.3 | 4.6 ± 2.68 | 1.3 ± 0.46 |

**Table 4:** Mean relative peptide amount in % measured in supernatant after 1 and 7 days peptide hydrogel incubation in PBS and medium.

|  | **P11-4** | | | **P11-8** | | **P11-13/14** | | **P11-28/29** | |
| --- | --- | --- | --- | --- | --- | --- | --- | --- | --- |
|  | 1 d | 7 d | | 1 d | 7 d | 1 d | 7 d | 1 d | 7 d |
| PBS | 10.6± 5.7 | | 9.4± 4.4 | 7.5± 4.3 | 6.9± 1.2 | 9.2± 4.9 | 12.0± 5.6 | 3.4± 0.4 | 6.6± 2.8 |
| Medium | 0.4± 0.1 | | 4.6± 2.5 | 3.8± 0.6 | 6.9 ± 5.8 | 1.3± 0.3 | 6.8± 3.7 | 0.4±0.1 | 2.2± 1.8 |

**Table 5:** Mean relative peptide amount in %, measured in the supernatant after 1 and 7 days of peptide hydrogel incubation in PBS and human neutrophil elastase(HNE) supplemented PBS (100 ug/ml).

|  | **P11-4** | | | **P11-8** | | **P11-13/14** | | **P11-28/29** | |
| --- | --- | --- | --- | --- | --- | --- | --- | --- | --- |
|  | 1 d | 7 d | | 1 d | 7 d | 1 d | 7 d | 1 d | 7 d |
| PBS | 10.6± 5.7 | | 9.4± 4.4 | 7.5± 4.3 | 6.9± 1.2 | 9.2± 4.9 | 14.0± 5.6 | 3.4± 0.4 | 6.6± 2.8 |
| HNE | 14.7± 2.8 | | 12.8± 5.8 | 10.4± 3.9 | 10.4± 1.3 | 13.0± 2.1 | 12.2± 5.2 | 4.2± 0.2 | 5.6± 0.7 |

**Table 5:** Mean relative peptide amount in %, measured in the supernatant after 1 and 7 days of peptide hydrogel incubation in medium and with different bacterial strains.

|  | **P11-4** | | | **P11-8** | | **P11-13/14** | | **P11-28/29** | |
| --- | --- | --- | --- | --- | --- | --- | --- | --- | --- |
|  | 1 d | 7 d | | 1 d | 7 d | 1 d | 7 d | 1 d | 7 d |
| Medium | 0.4± 0.1 | | 4.6± 2.5 | 3.8± 0.6 | 6.9± 5.8 | 1.3± 0.3 | 6.8± 4.7 | 0.4± 0.1 | 2.2± 1.8 |
| S.mutans | 3.4± 3.1 | | 10.8± 7.8 | 5.8± 3.7 | 8.5± 3.2 | 1.8± 1.4 | 6.8± 0.2 | 3.4± 3.1 | 4.5± 2.1 |
| S.aureus | 2.2± 1.8 | | 11.0± 7.5 | 7.9± 3.9 | 8.3± 7.4 | 1.9± 0.8 | 7.6± 3.1 | 2.2± 1.8 | 6.5± 4.5 |
| Paeruginosa | 1.6± 0.7 | | 12.2± 6.9 | 10.2± 0.3 | 12.3± 3.0 | 1.6± 0.3 | 8.3± 1.7 | 1.6± 0.7 | 2.8± 1.5 |
